# Rural-urban disparities in age trajectories of depression caseness in later life: The China Health and Retirement Longitudinal Study

**DOI:** 10.1371/journal.pone.0215907

**Published:** 2019-04-25

**Authors:** Yaoyue Hu, Peng Li, Pekka Martikainen

**Affiliations:** 1 Laboratory of Population Health, Max Planck Institute for Demographic Research, Rostock, Germany; 2 Population Research Unit, Faculty of Social Sciences, University of Helsinki, Helsinki, Finland; 3 Centre for Health Equity Studies (CHESS), Stockholm University and Karolinska Institutet, Stockholm, Sweden; VU Medisch Centrum School of Medical Sciences, NETHERLANDS

## Abstract

**Background:**

No consensus has been reached on whether depression decreases or increases with age in later life. Majority of the evidence comes from Western societies, while little is known about this relationship and its rural-urban disparities in the Chinese context.

**Methods:**

Three waves of data from 15,501 Chinese adults aged 45–85 years from the China Health and Retirement Longitudinal Study, Chinese sister study of Health and Retirement Study, were used. Depression caseness was identified using the 10-item Center for Epidemiologic Studies Depression Scale (score ≥12). Urbanisation levels were determined by combining rural-urban residence and rural-urban *Hukou* (a household registration system). Odds ratios and predicted probabilities of depression caseness were estimated using generalised linear mixed models.

**Results:**

For both men and women and across all ages, the crude predicted probability of depression caseness was the highest in the rural group, followed by the semi-urban group, and the lowest in the urban group. The probability was stable over age among urban men (around 0.05), but it increased at an accelerated rate with age among semi-urban men (0.25 at age 85, 95% confidence interval [CI]: 0.13–0.44) and rural men (0.29 at age 85, 95% CI: 0.22–0.39). Among women the age pattern was similar between the urbanisation groups: the probability increased with age, reached a peak at ages 75–80 (urban women: 0.16, 95% CI: 0.13–0.20; semi-urban women: 0.28, 95% CI: 0.20–0.39; rural women: 0.41, 95% CI: 0.36–0.46), and decreased slightly afterwards. These differences were significantly attenuated when socio-demographic characteristics and physical disability, but not when behaviour-related factors, were controlled for.

**Conclusion:**

The age trajectories of later-life depression caseness varied by gender and urbanisation levels, and were not U-shaped as in many Western societies. The increasing depression caseness with age and the large rural disadvantage were substantially driven by socio-demographic characteristics and physical disability.

## Introduction

Depression, China’s fourth leading cause of disability,[1] has been increasingly recognised as a serious public health concern in its older population.[2, 3] According to a recent meta-analysis of 32 studies, the prevalence of depressive symptoms was approximately 10% higher among rural older Chinese aged 60 and over (29.2%) than among their urban counterparts (20.5%).[4] This rural-urban disparity contradicts observations from Western societies or other eastern Asian countries where no substantial rural-urban difference or even an urban disadvantage was found.[5]

Older age can bring both strengths and vulnerabilities to mental health. Several strengths acquired over the life course could lead to decreasing level of depression in later life, such as improved general skills and strategies to avoid and solve problems and to manage unpredictable life events, more harmonious social and psychological traits, and better regulation of everyday emotion.[6–8] On the other hand, older age is also associated with declines in health, in physiological flexibility to cope with stressors, in sense of control over life, and in size of social network, which may jeopardise mental health.[6, 8]

No consensus has been reached on whether depression decreases or increases with age.[9, 10] A recent meta-analysis of cross-sectional studies from China showed that the prevalence of depressive symptoms increased from 22%, 25%, to 30% among Chinese adults aged 60–69, 70–79, and 80 and older, respectively.[11] In a prospective study of Canadians aged 65 and beyond, the probability of major depression was found to follow a U-shaped age trajectory with the nadir at approximately age 80.[12] Two longitudinal studies of Americans aged 18 and over also reported a U-shaped age pattern that the lowest level of depressive symptoms was found at around age 50[13] and 60[14], respectively. On the contrary, a prospective study of Germans aged 54–85 indicated that depressive symptoms were stable until approximately age 70 and increased for ages beyond.[15] Another prospective study of Americans aged 65 and older showed that, when socio-demographic characteristics, social support, and health status were controlled for, the linear age-related increase of depressive symptoms reversed and became decreasing with age.[16]

To our knowledge, no previous study has used longitudinal data to examine how depression changes with age in later life in the Chinese context, and how it differs for rural and urban Chinese. Additionally, Li and colleagues[5] criticised that most previous studies from China did not adjust for necessary covariates when examining the rural-urban disparities in late-life depression. In this study we employed a large national representative sample of middle-aged and older Chinese adults to investigate 1) how depression caseness changed with increasing age; 2) the rural-urban difference in this age pattern of depression caseness; and 3) the contributions of socio-demographic characteristics, behaviour-related factors, and physical disability to the rural-urban difference.

## Methods

### Study design

We used longitudinal data from the nationally representative China Health and Retirement Longitudinal Study (CHARLS)–a sister study of the U.S. Health and Retirement Study (HRS)–of Chinese community-dwelling adults aged 45 and older and their spouses.[17] The CHARLS was approved by the Ethical Review Committee at Peking University, and all the participants signed a written informed consent.[18] Participants were randomly drawn from households from 450 villages/neighbourhoods of 150 counties, covering 28 provinces. The baseline survey (Wave 1) was conducted in 2011/12, and two biannual follow-up surveys (Waves 2 & 4) were carried out in 2013 and 2015, respectively. The response rate for Waves 1 was 80.5%,[17] and the follow-up rate for Waves 2 and 4 was 85.8% and 82.3%, respectively. Comprehensive information on socio-demographic characteristics, household, physical and mental health status, functioning, and lifestyles were collected. Details of the CHARLS can be found elsewhere.[17] We included 7,334 men and 8,167 women aged 45–85 at any given wave and for whom a depression scale could be calculated (88% of the total sample).

### Urbanisation

Whether the village/neighbourhood where the participants’ household was living in was rural or urban was determined in accordance with the National Bureau of Statistics China. Given China’s profound rural-urban segregation associated with the *Hukou* system (a household registration system),[19] combining the *Hukou* status and place of residence measured at Wave 1, we derived three urbanisation groups: rural (rural *Hukou* holders living in rural areas), semi-urban (rural *Hukou* holders living in urban areas), and urban (urban *Hukou* holders).

### Depression caseness

At each wave, the 10-item Center for Epidemiologic Studies Depression Scale (CES-D-10) was administrated to measure the frequency of depressive symptoms in the past week (rarely or none, some days, occasionally, and most of the time coded as 0–3, reversed for positive items).[20] The CES-D-10 questionnaire was validated previously in Chinese elderly.[21] We calculated the CES-D-10 score (0–30) among participants who responded to at least nine items.[20] For those with one item missing its value was imputed by the mean of the participant’s non-missing items. We defined depression caseness as a CES-D-10 score higher than a cut-off threshold of 12. This has been validated to denote clinically significant depressive symptoms in an older Chinese population.[22]

### Socio-demographic factors

Four regions of residence (East, Northeast, Central, and West China) was grouped in line with the National Bureau of Statistics China. Educational attainment (no formal education, primary, lower secondary, and upper secondary or higher education) was measured at Wave 1. Number of household amenities (e.g., automobile, refrigerator, television, and mobile phone; maximum 17 amenities), retirement status (yes/no), and marital status were measured at all waves. Marital status was dichotomised into married and unmarried (separated, divorced, widowed, and never married).

### Behaviour-related factors

Behaviour-related factors were measured at all waves as well. Based on the beverage-specific frequency of drinking beer, wine/rice wine, and liquor in the past year, we obtained the maximum frequency of drinking any beverage (men: no, <1/month, 1-3/month, 1-6/week, 1+/day; women: no/year). Smoking status differed for men (never, former, and current) and women (never/ever). Body mass index (BMI) was derived from objectively measured height (m) and weight (kg), and further categorised using cut-off thresholds for Chinese population (underweight: <18.5, normal weight: 18.5–23.9, overweight: 24.0–27.9, obese: ≥28.0).[23] Frequency (none, not regularly, almost every week, and almost daily) of engaging in various social activities in the past month (e.g., interacting with friends, and going to community club) was also assessed.

### Physical disability

Physical disability was repeatedly assessed at all waves by the number of limitations in activities of daily living (ADLs) including dressing, bathing or showing, eating, getting into or out of bed, using toilet, and controlling urination and defecation.

### Statistical analysis

The relationship between age and depression caseness was analysed using generalised linear mixed models (GLMMs), which account for dependency of repeated observations within individuals and accommodate for binominal response.[24] Age and age squared were entered in models to capture possibly curvilinear age trajectories. All the analyses were stratified by gender as we found an interaction between age and gender (p<0.001). We used the random slope model allowing the slope of age to vary by participants as it was favoured over the random intercept model (women: p<0.01, men: p = 1.00). Evidence was found for period effects (waves; p<0.001 for both genders) but not for cohort effects (birth years; women: p = 0.06, men: p = 0.77), thus we kept period dummies in the final models. In addition, we tested for the interactions between age and urbanisation (women: p = 0.46, men: p = 0.001), between age squared and urbanisation (women: p = 0.47, men: p<0.01), and between age and period (women: p<0.05, men: p = 0.60). These interactions were thus included in our final models, and allowed the trajectories of depression caseness to have a different slope over age and a different age pattern for each urbanisation level.

We first estimated the crude association between age and depression caseness and its rural-urban difference (Model 1), and then adjusted for socio-demographic factors (Model 2), and additionally for behavioural-related factors (Model 3) and physical disability (Model 4). Odds ratios were presented, but marginal predicted probabilities were also extracted and plotted. Inverse probability weights (IPWs) were calculated and applied to account for baseline non-response (20%) and missing CES-D-10 score at Wave 1 (11%).[18] Missingness in covariates, which mainly came from BMI due to the non-participation of anthropometric measurements (21%), was coded as a separate category for each covariate. Missing CES-D-10 score at follow-up was handled by GLMMs, a technique uses all available information of incomplete longitudinal data and is valid under missing at random (missingness depends only on observed data).[25] Sensitivity analyses were performed among participants with non-missing BMI at Wave 1, in which another set of IPWs additionally accounting for the non-participation of anthropometric measurements at Wave 1 was generated and used. All statistical analyses were performed separately for men and for women, using *R* package ‘lme4’ in *RStudio* version 1.0.143 (Rstudio, 2015, Boston).

## Results

More than half of our sample were rural *Hukou* holders living in rural areas; whereas the share of rural and urban *Hukou* holders living in urban areas was similar (Table 1). Depression caseness was more common among the less urbanised groups and among women. Urban men and women also differed from their less urbanised counterparts that they received more education, were retired, had more household amenities, and engaged in social activities more often. Additionally, more urban men were overweight and obese but less of them were current smokers than semi-urban and rural men.

**Table 1:** Distribution of depression caseness, socio-demographic factor, behavioural-related factors, and physical disability at CHARLS Wave 1, by urbanisation groups and gender.

	Men (%)			Women (%)		
	Rural	Semi-rural	Urban	Rural	Semi-rural	Urban
**Total number of participants**	4176	1375	1783	4739	1701	1727
**Total number of observations for all waves**	10634	3380	4350	11988	4202	4091
**Depression caseness**						
No	73.6	80.3	86.8	60.2	69.2	79.0
Yes	26.4	19.7	13.2	39.8	30.8	21.0
**Socio-demographic factors**						
***Region***						
East	30.1	41.3	28.9	29.7	37.6	28.4
Central	29.7	25.4	28.3	29.9	25.9	26.4
West	34.5	30.8	28.0	34.5	33.7	27.9
Northeast	5.7	2.5	14.8	5.9	2.9	17.3
***Age***						
<55	34.0	41.0	29.7	39.6	43.0	38.7
55–64	39.1	37.2	37.4	36.5	35.2	34.7
65–74	19.7	17.0	23.3	17.2	15.5	19.2
≥75	7.2	4.8	9.6	6.6	6.4	7.5
***Marital status***						
Married	85.8	89.3	90.9	79.6	79.9	79.8
Unmarried	14.3	10.7	9.1	20.5	20.1	20.2
***Education***						
No formal education	37.9	32.2	13.0	69.5	58.8	25.0
Primary	28.4	28.6	19.0	16.5	19.8	15.8
Lower secondary	24.7	28.5	27.8	11.2	16.5	28.3
Upper secondary or higher	8.7	10.5	39.8	2.7	4.7	30.6
Missing	0.3	0.2	0.5	0.1	0.2	0.4
***Retired***						
No	85.6	78.2	50.9	77.3	66.4	37.7
Yes	14.1	21.2	48.4	22.5	33.0	61.5
Missing	0.3	0.7	0.7	0.2	0.6	0.8
***Number of household amenities***						
0–2	30.4	18.8	11.8	29.3	18.9	12.5
3–4	37.2	30.3	29.1	37.4	30.5	29.4
5–6	23.6	30.7	32.1	23.6	29.8	30.6
≥7	8.5	19.5	24.6	9.0	19.8	25.1
Missing	0.4	0.7	2.4	0.8	0.9	2.3
**Behavioural-related factors**						
***Social activities***						
No	53.0	53.1	40.8	52.2	54.5	44.0
Less often	16.1	13.9	13.6	13.9	11.9	10.7
Almost every week	12.2	12.8	15.7	10.2	10.2	12.5
Almost daily	18.7	20.1	29.9	23.6	23.4	33.0
Missing	0.1	0.2	0.1	0.1	0	0
***Alcohol drinking frequency***						
No	43.9	42.6	46.4	87.9	88.8	89.0
Yes				9.5	9.0	9.0
<1/month	10.5	11.2	10.8			
1-3/month	6.4	6.3	6.0			
1-6/week	9.3	9.2	10.3			
1+/day	21.9	23.9	17.4			
Missing	7.9	6.8	9.1	2.6	2.2	2.0
***Smoking***						
Never	23.8	25.4	31.9	91.9	90.8	93.5
Former/current				8.2	9.2	6.5
Former	14.9	15.7	20.2			
Current	61.3	58.9	48.0			
Missing	0.1	0	0	0	0	0
***BMI***						
<18.5	7.0	5.0	3.1	7.4	4.0	2.5
18.5–23.9	57.3	46.6	33.7	43.5	37.8	28.8
24.0–27.9	18.8	23.9	26.8	26.6	28.5	26.5
≥28.0	5.3	8.4	9.0	10.9	14.6	12.9
Missing	11.6	16.3	27.5	11.7	15.1	29.2
**ADL limitations**						
0	83.4	87.7	87.9	78.2	82.8	88.5
1	8.2	6.0	6.9	10.1	8.1	5.5
2+	7.9	5.2	4.4	10.6	8.4	5.3
Missing	0.6	1.1	0.8	1.2	0.7	0.7

**Fig 1:**
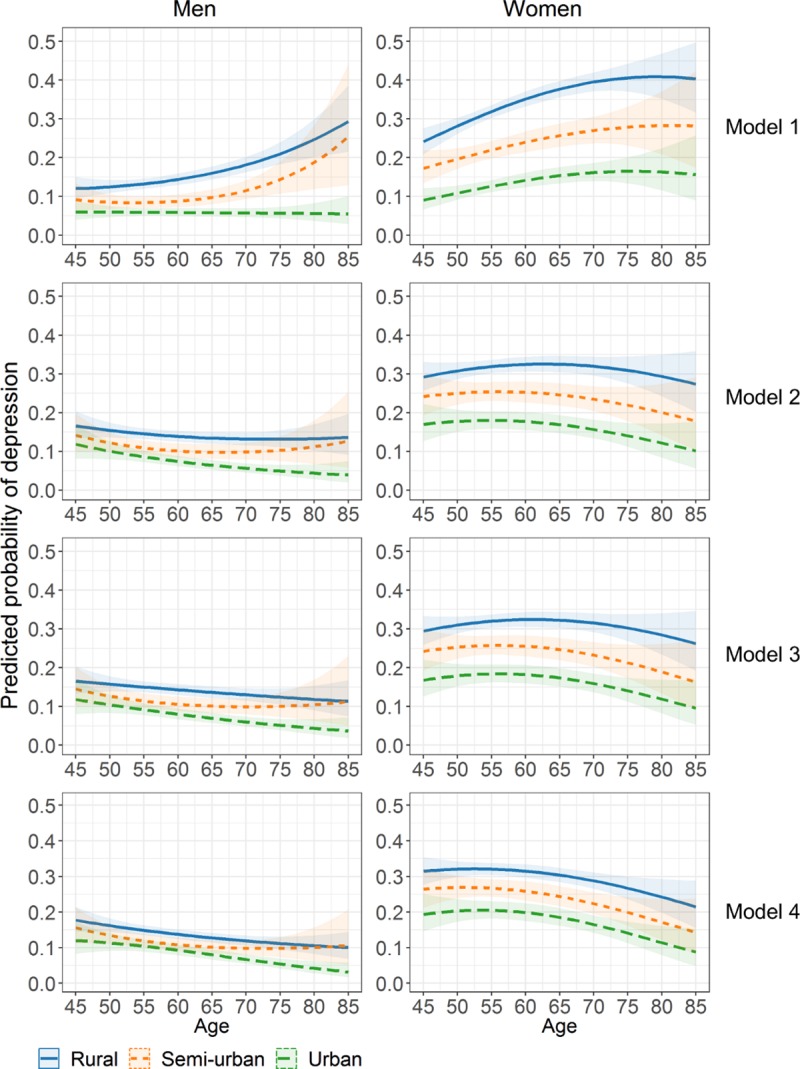
Rural-urban difference in age trajectories of the marginal predicted probability of depression caseness.

Tables 2 and 3 provide results on the association between age and depression caseness and its rural-urban difference for men and women, respectively. The predicted probabilities of depression caseness over age are plotted in Fig 1. Because of the interactions of age and age squared with urbanisation levels, Fig 1 is a more straightforward way to present the rural-urban difference in predicted probabilities in depression caseness over ages 45–85 with their 95% confidence intervals. For both genders and over all ages, the crude probability of depression caseness (Model 1) was the highest in the rural group, followed by the semi-urban group, and the lowest in the urban group. Compared to men, the crude probability was much higher and the rural-urban disparities were much more evident among women. The crude probability was stable over age among urban men (probability 0.05–0.06). However the crude probability increased at an accelerated rate with age from 0.09 at age 45 (95% confidence interval [CI]: 0.06–0.13) to 0.25 at age 85 (95% CI: 0.13–0.44) among semi-urban men, and from 0.12 (95% CI: 0.10–0.15) to 0.29 (95% CI: 0.22–0.39) among rural men, respectively. Among women, the age trajectories were similar between urbanisation groups: the crude probability increased from age 45 (urban women: 0.09, 95% CI: 0.07–0.12; semi-urban women: 0.17, 95% CI: 0.13–0.22; rural women: 0.24, 95% CI: 0.21–0.28), hit the peak at age 76 for urban women (0.16, 95% confidence interval [CI]: 0.13–0.20), age 82 for semi-urban women (0.28, 95% CI: 0.20–0.39), and age 79 for rural women (0.41, 95% CI:0.36–0.46), and decreased slightly afterwards.

**Table 2:** Association between age and depression caseness and its rural-urban difference among men.

	Model 1	Model 2	Model 3	Model 4
	OR (95% CI)	OR (95% CI)	OR (95% CI)	OR (95% CI)
**Age** (centred at 45 years)	1.01 (0.98,1.04)	0.99 (0.96,1.02)	1.00 (0.97,1.03)	0.99 (0.96,1.01)
**Age**^**2**^ (centred at 45 years)	1.00 (1.00,1.00)	1.00 (1.00,1.00)	1.00 (1.00,1.00)	1.00 (1.00,1.00)
**Urbanisation**				
Rural	Ref	Ref	Ref	Ref
Semi-urban	0.74 (0.47,1.16)	0.83 (0.54,1.28)	0.86 (0.55,1.32)	0.86 (0.56,1.31)
Urban	0.46 (0.29,0.73)	0.67 (0.43,1.06)	0.68 (0.43,1.06)	0.63 (0.41,0.99)
**Region**				
East		Ref	Ref	Ref
Central		1.64 (1.40,1.92)	1.61 (1.38,1.88)	1.48 (1.27,1.72)
Northeast		1.11 (0.85,1.43)	1.14 (0.88,1.73)	0.98 (0.76,1.26)
West		1.78 (1.53,2.07)	1.71 (1.47,1.99)	1.60 (1.38,1.86)
**Education**				
No formal education		Ref	Ref	Ref
Primary		0.80 (0.68,0.93)	0.81 (0.70,0.95)	0.86 (0.74,1.00)
Lower secondary		0.68 (0.57,0.80)	0.71 (0.60,0.84)	0.78 (0.66,0.91)
Upper secondary & higher		0.56 (0.45,0.70)	0.59 (0.47,0.73)	0.66 (0.53,0.81)
Missing		0.83 (0.32,2.18)	0.78 (0.31,2.01)	0.94 (0.38,2.34)
**Retirement**				
No		Ref	Ref	Ref
Yes		1.40 (1.21,1.61)	1.35 (1.17,1.55)	1.13 (0.98,1.30)
Missing		1.70 (0.94,3.05)	1.69 (0.95,3.01)	1.46 (0.81,2.62)
**Marital status**				
Married		Ref	Ref	Ref
Unmarried		1.54(1.29,1.84)	1.55 (1.30,1.84)	1.58 (1.34,1.87)
**Household amenities**				
0–2		Ref	Ref	Ref
3–4		0.76 (0.65,0.88)	0.78 (0.67,0.90)	0.81 (0.70,0.94)
5–6		0.50 (0.42,0.59)	0.53 (0.45,0.62)	0.56 (0.48,0.66)
≥7		0.36 (0.30,0.44)	0.39 (0.32,0.48)	0.43 (0.35,0.52)
Missing		0.70 (0.41,1.20)	0.70 (0.41,1.19)	0.68 (0.40,1.15)
**Social activity**				
No			Ref	Ref
Less often			0.97 (0.84,1.12)	0.98 (0.85,1.13)
Almost every week			0.70 (0.59,0.83)	0.72 (0.61,0.85)
Almost daily			0.68 (0.59,0.79)	0.72 (0.63,0.83)
Missing			1.65 (0.28,9.71)	1.50 (0.26,8.65)
**Alcohol drinking**				
No			Ref	Ref
<1/month			0.83 (0.69,0.99)	0.86 (0.72,1.03)
1-3/month			0.88 (0.72,1.07)	0.88 (0.72,1.08)
1-6/week			0.89 (0.74,1.07)	0.90 (0.75,1.08)
≥1/day			0.68 (0.59,0.78)	0.72 (0.63,0.83)
Missing			0.66 (0.49,0.89)	0.66 (0.49,0.89)
**Smoking**				
Never			Ref	Ref
Former			1.32 (1.11,1.57)	1.23 (1.03,1.46)
Current			1.26 (1.08,1.47)	1.26 (1.08,1.46)
Missing			1.12 (0.90,1.40)	1.12 (0.90,1.39)
**BMI**				
>15 & ≤18.5			1.45 (1.15,1.83)	1.41 (1.12,1.76)
>18.5 & ≤24			Ref	Ref
>24 & ≤28			0.85 (0.74,0.98)	0.80 (0.69,0.92)
>28			0.77 (0.61,0.97)	0.70 (0.55,0.88)
Missing			1.05 (0.92,1.21)	1.00 (0.87,1.15)
**Number of ADLs**				
0				Ref
1				2.75 (2.32,3.26)
2+				6.44 (5.36,7.74)
Missing				0.42 (0.19,0.91)
**Age × Urbanisation**				
Age × semi-urban	0.97 (0.91,1.03)	1.02 (0.96,1.09)	0.98 (0.92,1.04)	0.98 (0.93,1.04)
Age × urban	0.99 (0.94,1.05)	0.96 (0.90,1.02)	0.98 (0.93,1.04)	1.01 (0.96,1.07)
Age^2^ × semi-urban	1.00 (1.00,1.00)	1.00 (1.00,1.00)	1.00 (1.00,1.00)	1.00 (1.00,1.00)
Age^2^ × urban	1.00 (1.00,1.00)	1.00 (1.00,1.00)	1.00 (1.00,1.00)	1.00 (1.00,1.00)
**Period**				
Wave 1	Ref	Ref	Ref	Ref
Wave2	0.81 (0.65,1.01)	1.04 (0.80,1.37)	1.06 (0.84,1.35)	1.01 (0.80,1.29)
Wave3	0.89 (0.70,1.14)	1.13 (0.85,1.52)	1.15 (0.89,1.47)	1.12 (0.87,1.43)
**Age × Period**				
Age×Wave2	0.99 (0.98,1.00)	0.98 (0.97,1.00)	0.98 (0.97,1.00)	0.99 (0.97,1.00)
Age×Wave3	0.99 (0.98,1.01)	0.99 (0.97,1.00)	0.99 (0.97,1.00)	0.99 (0.97,1.00)

OR: odds ratio; CI: confidence interval; Ref: reference category

**Table 3:** Association between age and depression caseness and its rural-urban difference among women.

	Model 1	Model 2	Model 3	Model 4
	OR (95% CI)	OR (95% CI)	OR (95% CI)	OR (95% CI)
**Age** (centred at 45 years)	1.05 (1.03,1.08)	1.03 (1.00,1.05)	1.03 (1.00,1.05)	1.02 (0.99,1.04)
**Age**^**2**^ (centred at 45 years)	1.00 (1.00,1.00)	1.00 (1.00,1.00)	1.00 (1.00,1.00)	1.00 (1.00,1.00)
**Urbanisation**				
Rural	Ref	Ref	Ref	Ref
Semi-urban	0.66 (0.47,0.92)	0.77 (0.56,1.07)	0.76 (0.55,1.06)	0.78 (0.57,1.08)
Urban	0.31 (0.22,0.45)	0.50 (0.34,0.71)	0.48 (0.34,0.70)	0.52 (0.36,0.75)
**Region**				
East		Ref	Ref	Ref
Central		1.64 (1.45,1.86)	1.65 (1.45,1.87)	1.54 (1.36,1.74)
Northeast		1.39 (1.14,1.69)	1.34 (1.10,1.64)	1.27 (1.04,1.54)
West		1.98 (1.75,2.24)	1.95 (1.72,2.21)	1.80 (1.60,2.04)
**Education**				
No formal education		Ref	Ref	Ref
Primary		0.79 (0.69,0.90)	0.80 (0.69,0.93)	0.82 (0.72,0.94)
Lower secondary		0.69 (0.59,0.80)	0.69 (0.55,0.77)	0.74 (0.63,0.85)
Upper secondary & higher		0.47 (0.38,0.57)	0.48 (0.41,0.69)	0.50 (0.40,0.61)
Missing		0.40 (0.11,1.41)	0.40 (0.11,1.41)	0.47 (0.14,1.62)
**Retirement**				
No		Ref	Ref	Ref
Yes		1.29 (1.17,1.43)	1.32 (1.19,1.46)	1.18 (1.07,1.31)
Missing		1.39 (0.78,2.48)	1.39 (0.78,2.49)	1.22 (0.68,2.18)
**Marital status**				
Married		Ref	Ref	Ref
Unmarried		1.18 (1.04,1.34)	1.19 (1.05,1.35)	1.17 (1.04,1.32)
**Household amenities**				
0–2		Ref	Ref	Ref
3–4		0.89 (0.79,1.01)	0.90 (0.79,1.02)	0.94 (0.83,1.06)
5–6		0.64 (0.56,0.74)	0.66 (0.58,0.75)	0.69 (0.60,0.79)
≥7		0.51 (0.43,0.59)	0.52 (0.44,0.61)	0.56 (0.48,0.65)
Missing		0.51 (0.34,0.76)	0.50 (0.34,0.75)	0.52 (0.35,0.77)
**Social activity**				
No			Ref	Ref
Less often			1.06 (0.94,1.21)	1.08 (0.95,1.22)
Almost every week			0.86 (0.74,0.99)	0.89 (0.77,1.03)
Almost daily			0.76 (0.69,0.85)	0.80 (0.72,0.89)
Missing			2.21 (0.38,12.99)	2.49 (0.41,15.23)
**Alcohol drinking**				
No			Ref	Ref
Yes			1.16 (1.02,1.33)	1.13 (0.99,1.29)
Missing			1.33 (0.90,1.95)	1.33 (0.90,1.95)
**Smoking**				
Never			Ref	Ref
Former/current			1.23 (1.03,1.48)	1.17 (0.98,1.40)
Missing			1.13 (0.76,1.67)	1.10 (0.74,1.62)
**BMI**				
>15 & ≤18.5			1.16 (0.94,1.43)	1.16 (0.94,1.42)
>18.5 & ≤24			Ref	Ref
>24 & ≤28			0.99 (0.88,1.10)	0.95 (0.85,1.06)
>28			0.92 (0.79,1.07)	0.82 (0.71,0.96)
Missing			1.02 (0.91,1.16)	0.97 (0.86,1.10)
**Number of ADLs**				
0				Ref
1				2.20 (1.93,2.51)
2+				4.73 (4.07,5.49)
Missing				0.35 (0.19,0.62)
**Age × Urbanisation**				
Age × semi-urban	0.99 (0.95,1.03)	0.99 (0.95,1.04)	1.00 (0.96,1.04)	1.00 (0.96,1.04)
Age × urban	1.02 (0.95,1.05)	1.02 (0.95,1.04)	1.03 (0.96,1.05)	1.01 (0.96,1.06)
Age^2^ × semi-urban	1.00 (1.00,1.00)	1.00 (1.00,1.00)	1.00 (1.00,1.00)	1.00 (1.00,1.00)
Age^2^ × urban	1.00 (1.00,1.00)	1.00 (1.00,1.00)	1.00 (1.00,1.00)	1.00 (1.00,1.00)
**Period**				
Wave 1	Ref	Ref	Ref	Ref
Wave2	0.87 (0.74,1.04)	1.04 (0.87,1.23)	1.04 (0.87,1.23)	1.02 (0.85,1.21)
Wave3	1.02 (0.84,1.23)	1.23 (1.02,1.49)	1.23 (1.02,1.49)	1.20 (0.99,1.46)
**Age × Period**				
Age×Wave2	0.99 (0.98,1.00)	0.98 (0.97,0.99)	0.98 (0.97,0.99)	0.99 (0.98,1.00)
Age×Wave3	0.99 (0.98,1.00)	0.99 (0.98,1.00)	0.99 (0.98,1.00)	0.99 (0.97,1.00)

OR: odds ratio; CI: confidence interval; Ref: reference category

Results from Model 2 adjusting for socio-demographic factors showed that, for both genders, a higher risk of depression caseness was associated with living in Central, Northeast, and West China vs. East China, being retired, being unmarried, and having less education and fewer household amenities. These socio-demographic factors substantially attenuated the rural-urban disparities in depression caseness over age (Fig 1, Model 2). Among rural and urban men, the probability of depression caseness became decreasing with age, while it was slightly U-shaped among semi-urban men. Among women, the age pattern was in an inversed U-shape in all urbanisation groups with the peak age at around 60 (56 for semi-urban women).

Further adjustment for behaviour-related factors (Model 3), among which, a lower risk of depression caseness was associated with engaging in social activity more frequently for both genders, drinking less than once per month or at least once a day among men, and being overweight and obese among men. A higher risk of depression caseness was associated former and/or current smoking for both genders, alcohol drinking among women, and being underweight among men. The age pattern of depression caseness and its rural-urban difference changed very slightly after further controlling for behavioural-related factors (Fig 1, Model 3).

Physical disability had a large effect on depression caseness (Model 4). Compared to no ADL limitation, one limitation was associated with 2.75 (95% CI:2.32–3.26) and 2.20 (95% CI: 1.93–2.51) times higher odds of depression caseness among men and women, respectively. The odds increased by four- to six-fold for having at least two limitations. The age trajectories of depression caseness, after adjusting for all sets of factors, became more similar between men and women and between urbanisation groups whereby the probability of depression caseness decreased after approximately age 55 (Fig 1, Model 4).

As shown in Fig 1, the age trajectories of depression caseness were different for rural, semi-urban, and urban women in all the models. Among men, the age trajectories of depression caseness differed by urbanisation levels in Model 1. However, in Model 4 the age trajectories were different between rural and urban men, but for semi-urban men the confident intervals overlapped with the other groups.

Results of the sensitivity analyses among participants with non-missing BMI at Wave 1, in general, were similar to those from the full sample (Table A, Table B, and Figure A in S1 File). The main differences were that, the crude probability of depression caseness (Model 1) increased more linearly with age for semi-urban and rural men; whereas the age pattern was in inversely U-shaped in all models among urban women.

## Discussion

In this study using a large nationally representative sample of middle-aged and older Chinese adults, we found that, across all ages, rural men and women had the highest probability of depression caseness, followed by the semi-urban ones, and the urban ones had the lowest probability. The age trajectories of depression caseness differed between urbanisation groups among men but were highly similar among women. The probability of depression caseness did not change with age among urban men, however it increased more rapidly at older ages among semi-urban and rural men. Among women, the probability increased with age at a decelerated rate with a peak at ages 75–80. After controlling for socio-demographic factors, the probability became decreasing with age. The rural-urban disparities in the age trajectories of depression caseness largely reduced when socio-demographic characteristics and physical disability were adjusted for, whereas behaviour-related factors only played a lesser role.

China has been urbanising rapidly with the proportion of urban population increasing from 26% in 1990 to 59% in 2017. Moving to urban areas is viewed as a means of upward social mobility in China: it is associated with better education, more economic resources, and more opportunities, and also with a sense of cultural and psychological pre-eminence.[19] However, the rural *Hukou* holders who migrate to cities are often a marginalised group in their urban residence.[26] They face social stigma and discrimination in job opportunities and wages in cities, as well as institutional exclusions from free education, social welfare, and health insurance, which are tied with their rural *Hukou*.[27] Such social stigma and discrimination towards them may form long-term social stress that undermines their mental health.[27, 28] For working-age rural *Hukou* holders who stayed in rural areas, staying may not be their voluntary decision due to various reasons such as ill health of themselves and/or of their elderly parents,[29] which can cause mental distress for them particularly when they compare themselves with those who migrate to cities. Rural elderly (e.g., aged 65 and over) are often empty-nest elderly who are “left behind” by their adult children who migrated to cities. These “left-behind” rural elderly have been shown to have more (severe) depressive symptoms than those whose adult children did not migrate to cities.[30]

We speculate that the semi-urban elderly move into cities to live closer to their adult children, in order to receive better care and support from the adult children, as well as to help taking care of the grandchildren. Their increased probability of depression may be a result of narrowed social network and lack of extended social support, in addition to the discrimination in their urban residence. However, social network and social support are different concepts than the social interaction that we controlled for in our models. Social support has been defined as all the available social resources that an individual perceives to have, from both formal and informal helping relationships.[31] Social support arises from one’s social network, which include close social ties such as the partner and other nuclear family members, as well as from more distant and role-defined social ties such as friends and relatives.[31] Older adults who moved to cities where their adult children live, may have lost their distant social ties and possibly close social ties (e.g., when their partner did not move with them), leading to reduced social support that could jeopardise mental health particularly during times of crisis.

We did not find a U-shaped age pattern of depression caseness,[13, 14] nor a linear increase with age,[12, 16] as previously reported in studies from Western societies. The age patterns were rather diverse among middle-aged and older Chinese. The accelerated increase in depression caseness with age among semi-urban and rural male elderly, which was very different from their urban and female counterparts, may be owing to greater difficulties and struggles for them to be “left-behind” in rural areas or to move to cities. Despite the huge rural-urban disparities in the level of mental health, the highly similar age trajectories between urbanisation groups among women, even after controlling for a number of covariates, suggests that the observed age patterns are relatively general in nature. These common age patterns could be driven by often experienced gender inequalities in education, employment, and income, and in division of family work, which have existed and still exist in China.[32, 33] The less education older Chinese women obtained compared to older men has been demonstrated to play a major role in the gender gap in self-rated health and cognition at older ages.[34, 35] The gender difference in the level of mental health could also be attributable to the reporting bias that women are more open to report negative feelings and symptoms associated with depression, whereas men are less likely to do so.[36]

The decreasing probability of depression caseness with age which only emerged after controlling for socio-demographic factors indicates that–rather than late-life depression being mainly driven by ill health and multi-morbidity[12]–it may be more related to socioeconomic position, region of residence, and marital status for middle-aged and older Chinese. The behaviour-related factors did not contribute to the age pattern of depression caseness nor the rural-urban difference in the age pattern, once we had taken socio-demographic factors already into account. This could be due to the relatively small rural-urban difference in the behaviour-related factors in our sample, or the difference is highly correlated with the socio-demographic factors. The protective effect of overweight and obesity for mental health we found was the opposite with a meta-analysis of studies from high-income Western countries[37] but in line with a study from Shanghai[38], which may be explained by the higher prevalence of overweight and obesity among urban Chinese because of more Westernised food and lack of physically demanding exercise.

Physical disability is a major risk factor of depression among middle-aged and older Chinese, particularly among rural and semi-urban elderly likely owing to the lack of social and health care provided by the state and the less resources they and their adult children have. The situation could be worse for empty-nest rural elderly who are “left behind” by their adult children–the main caregiver of their ill elderly parents in the Chinese culture. This interpretation may be partially supported by the fact that the suicide rate among Chinese rural elderly is 3–5 times higher than their urban counterparts.[39]

Our study has several strengths and limitations. This is the first study that, to our knowledge, used repeatedly measured data from a large nationally representative sample of middle-aged and older Chinese adults to examine the age trajectories of depression, its rural-urban difference, and the effects of various possible drivers. Our findings were based on a prospective cohort study of community-dwelling middle-aged and older Chinese adults. Although those who were living in residential homes or nursing homes were not specifically included in CHARLS, this is unlikely to affect our findings because it remains largely socially unacceptable to send the elderly to residential or nursing homes.[40] Our study extended previous cross-sectional studies using CHARLS data to assess the association of depressive symptoms by *Hukou* status[5, 41] or place of residence[42, 43]. We confirmed the importance of combining *Hukou* status and place of residence to better reflect urbanisation levels in Chinese population in relation to mental health. The depression caseness identified in our study was based on a self-reported questionnaire rather than from doctor diagnosis. Clinically diagnosed depression is substantially lower in China than in Western countries due to the social stigma attached to mental illness in the Chinese culture, and often the Chinese tend to deny depression or claim symptoms to be related to somatic conditions.[44] As a result, it is possible that depressive symptoms assessed by CES-D-10 questionnaire may capture the mental health of the Chinese population better than clinical examinations. It is nevertheless possible that we omitted certain determinants of depression among middle-aged and older Chinese, for example, psychological factors (e.g., stress), living arrangement (e.g., co-residence with adult children), and informal care giving (e.g., provision of care to the grandchildren) or care receipt. Although GLMMs provide unbiased estimates under missing at random, there could be some unobserved mechanisms of dropout and non-response to CES-D-10 questionnaire that bias our findings.

To sum up, older Chinese adults who are rural *Hukou* holder, older, retired, unmarried, physically disabled, have lower education and less physical wealth should be targeted to reduce the disease burden of depression in China’s middle-aged and older population. Our findings also underline the importance of providing better mental health care in rural China where lack of mental health professionals and care facilities is pressing.[45]

## Supporting information

S1 File (DOCX)

## References

[1] GBD 2016 Disease and Injury Incidence and Prevalence Collaborators. Global, regional, and national incidence, prevalence, and years lived with disability for 328 diseases and injuries for 195 countries, 1990–2016: a systematic analysis for the Global Burden of Disease Study 2016. Lancet. 2017;390(10100):1211–59. 10.1016/S0140-6736(17)32154-2 28919117 PMC5605509

[2] World Health Organization. China country assessment report on ageing and heatlh. Geneva, Switzerland: 2015.

[3] Yang GH, Wang Y, Zeng YX, Gao GF, Liang XF, Zhou MG, et al Rapid health transition in China, 1990–2010: findings from the Global Burden of Disease Study 2010. Lancet. 2013;381(9882):1987–2015. 10.1016/S0140-6736(13)61097-1 23746901 PMC7159289

[4] Zhang L, Xu Y, Nie HW, Zhang YD, Wu Y. The prevalence of depressive symptoms among the older in China: a meta-analysis. Int J Geriatr Psych. 2012;27(9):900–6. 10.1002/gps.2821 22252938

[5] Li LW, Liu JY, Xu HW, Zhang ZM. Understanding Rural-Urban Differences in Depressive Symptoms Among Older Adults in China. J Aging Health. 2016;28(2):341–62. 10.1177/0898264315591003 26100620 PMC4893784

[6] Ross CE, Mirowsky J. Age and the balance of emotions. Soc Sci Med. 2008;66(12):2391–400 10.1016/j.socscimed.2008.01.048 18339465

[7] Mirowsky J, Ross CE. Age and Depression. J Health Soc Behav. 1992;33(3):187–205. 1401846

[8] Charles ST. Strength and vulnerability integration: a model of emotional well-being across adulthood. Psychol Bull. 2010;136(6):1068–91. 10.1037/a0021232 21038939 PMC3059514

[9] Riedel-Heller SG, Busse A, Angermeyer MC. The state of mental health in old-age across the 'old' European Union: A systematic review. Acta Psychiat Scand. 2006;113(5):388–401. 10.1111/j.1600-0447.2005.00632.x 16603030

[10] Jorm AF. Does old age reduce the risk of anxiety and depression? A review of epidemiological studies across the adult life span. Psychol Med. 2000;30(1):11–22. 10722172 10.1017/s0033291799001452

[11] Li D, Zhang DJ, Shao JJ, Qi XD, Tian L. A meta-analysis of the prevalence of depressive symptoms in Chinese older adults. Arch Gerontol Geriat. 2014;58(1):1–9. 10.1016/j.archger.2013.07.016 24001674

[12] Wu Z, Schimmele CM, Chappell NL. Aging and Late-Life Depression. J Aging Health. 2012;24(1):3–28. 10.1177/0898264311422599 21956098

[13] Mirowsky J, Kim JY. Graphing age trajectories—Vector graphs, synthetic and virtual cohort projections, and cross-sectional profiles of depression. Sociol Method Res. 2007;35(4):497–541.

[14] Sutin AR, Terracciano A, Milaneschi Y, An Y, Ferrucci L, Zonderman AB. The Trajectory of Depressive Symptoms Across the Adult Life Span. JAMA Psychiat. 2013;70(8):803–11. 10.1001/jamapsychiatry.2013.193 PMC3740038 23760442

[15] Rothermund K, Brandtstadter J. Depression in later life: Cross-sequential patterns and possible determinants. Psychol Aging. 2003;18(1):80–90. 12641314 10.1037/0882-7974.18.1.80

[16] Yang Y. Is old age depressing? Growth trajectories and cohort variations in late-life depression. J Health Soc Behav. 2007;48(1):16–32. 10.1177/002214650704800102 17476921

[17] Zhao Y, Hu Y, Smith JP, Strauss J, Yang G. Cohort profile: the China Health and Retirement Longitudinal Study (CHARLS). Int J Epidemiol. 2014;43(1):61–8. 10.1093/ije/dys203 23243115 PMC3937970

[18] Zhao Y, Strauss J, Yang G, Giles J, Hu P, Hu Y, et al China Health and Retirement Longitudinal Study– 2011–2012 national basleine users' guide 2013 16 7 2018 Available from: http://charls.pku.edu.cn/uploads/document/2011-charls-wave1/application/CHARLS_nationalbaseline_users_guide.pdf.

[19] Yang F, Lou VWQ. Childhood adversities, urbanisation and depressive symptoms among middle-aged and older adults: Evidence from a national survey in China. Ageing Soc. 2016;36(5):1031–51.

[20] Andresen EM, Malmgren JA, Carter WB, Patrick DL. Screening for depression in well older adults: Evaluation of a Short form of the CES-D. Am J Prev Med. 1994;10(2):77–84. 8037935

[21] Boey KW. Cross-validation of a short form of the CES-D in Chinese elderly. Int J Geriatr Psych. 1999;14(8):608–17. 10.1002/(sici)1099-1166(199908)14:8<608::aid-gps991>3.0.co;2-z 10489651

[22] Cheng ST, Chan ACM. The Center for Epidemiologic Studies Depression Scale in older Chinese: thresholds for long and short forms. Int J Geriatr Psych. 2005;20(5):465–70. 10.1002/gps.1314 15852439

[23] Zhou B. [Predictive values of body mass index and waist circumference to risk factors of related diseases in Chinese adult population]. Zhonghua liu xing bing xue za zhi = Zhonghua liuxingbingxue zazhi. 2002;23(1):5–10. 12015100

[24] Dean CB, Nielsen JD. Generalized linear mixed models: a review and some extensions. Lifetime Data Anal. 2007;13(4):497–512. 10.1007/s10985-007-9065-x 18000755

[25] Jansen I, Beunckens C, Molenberghs G, Verbeke G, Mallinckrodt C. Analyzing incomplete discrete longitudinal clinical trial data. Stat Sci. 2006;21(1):52–69.

[26] Wu XG, Treiman DJ. The household registration system and social stratification in China: 1955–1996. Demography. 2004;41(2):363–84. 15209045 10.1353/dem.2004.0010

[27] Li X, Stanton B, Fang X, Lin D. Social stigma and mental health among rural-to-urban migrants in China: A conceptual framework and future research needs. World Health Popul. 2006;8(3):14–31. 18277106 10.12927/whp.2006.18282 PMC2249560

[28] Aneshensel CS. Social stress: Theory and research. Annu Rev Sociol. 1992;18:15–38.

[29] Giles J, Mu R. Elderly parent health and the migration decisions of adult children: Evidence from rural China. Demography. 2007;44(2):265–88. 17583305 10.1353/dem.2007.0010

[30] Song Q. Facing "Double Jeopardy"? Depressive Symptoms in Left-Behind Elderly in Rural China. J Aging Health. 2017;29(7):1182–213. 10.1177/0898264316659964 27435491

[31] Gottlieb BH, Bergen AE. Social support concepts and measures. J Psychosom Res. 2010;69(5):511–20. 10.1016/j.jpsychores.2009.10.001 20955871

[32] Pearson V. Goods on which one loses: Women and mental health in China. Soc Sci Med. 1995;41(8):1159–73. 8578338 10.1016/0277-9536(94)00424-r

[33] Anson O, Sun SF. Gender and health in rural China: evidence from HeBei province. Soc Sci Med. 2002;55(6):1039–54. 12220088 10.1016/s0277-9536(01)00227-1

[34] Zhang H, d'Uva TB, van Doorslaer E. The gender health gap in China: A decomposition analysis. Econ Hum Biol. 2015;18:13–26. 10.1016/j.ehb.2015.03.001 25867249

[35] Lei X, Smith JP, Sun X, Zhao Y. Gender differences in cognition in China and reasons for change over time: Evidence from CHARLS. J Econ Ageing. 2014;4:46–55. 10.1016/j.jeoa.2013.11.001 25530942 PMC4269268

[36] Sonnenberg CM, Beekman ATF, Deeg DJH, van Tilburg W. Sex differences in late-life depression. Acta Psychiat Scand. 2000;101(4):286–92. 10782548

[37] Luppino FS, de Wit LM, Bouvy PF, Stijnen T, Cuijpers P, Penninx BWJH, et al Overweight, obesity, and depression: A systematic review and meta-analysis of longitudinal studies. Arch Gen Psychiat. 2010;67(3):220–9. 10.1001/archgenpsychiatry.2010.2 20194822

[38] Liu QL, Cai H, Yang LH, Xiang YB, Yang G, Li HL, et al Depressive symptoms and their association with social determinants and chronic diseases in middle-aged and elderly Chinese people. Sci Rep. 2018;8(1):3841 10.1038/s41598-018-22175-2 29497126 PMC5832867

[39] Li X, Xiao ZP, Xiao SF. Suicide among the elderly in mainland China. Psychogeriatrics. 2009;9(2):62–6. 10.1111/j.1479-8301.2009.00269.x 19604327

[40] Chen T. Living arrangement preferences and realities for elderly Chinese: implications for subjective wellbeing. Ageing Soc. 2018:1–25. 10.1017/S0144686X17001490 29422699 PMC5798623

[41] Guo J, Guan LD, Fang LM, Liu CC, Fu MQ, He H, et al Depression among Chinese older adults: A perspective from Hukou and health inequities. J Affect Disorders. 2017;223:115–20. 10.1016/j.jad.2017.07.032 28753468

[42] Lei XY, Sun XT, Strauss J, Zhang P, Zhao YH. Depressive symptoms and SES among the mid-aged and elderly in China: Evidence from the China Health and Retirement Longitudinal Study national baseline. Soc Sci Med. 2014;120:224–32. 10.1016/j.socscimed.2014.09.028 25261616 PMC4337774

[43] Xu YJ, Yang JJ, Gao JM, Zhou ZL, Zhang T, Ren JP, et al Decomposing socioeconomic inequalities in depressive symptoms among the elderly in China. BMC Public Health. 2016;16. 10.1186/s12889-016-3876-1 PMC5134228 27905918

[44] Parker G, Gladstone G, Chee KT. Depression in the planet's largest ethnic group: The Chinese. Am J Psychiat. 2001;158(6):857–64. 10.1176/appi.ajp.158.6.857 11384889

[45] Xiang YT, Ng CH, Yu X, Wang G. Rethinking progress and challenges of mental health care in China. World Psychiatry. 2018;17(2):231–2. 10.1002/wps.20500 29856546 PMC5980243

