# Supplementary material for: Rural-urban disparities in age trajectories of depression caseness in later life: The China Health and Retirement Longitudinal Study
Source: PLoS One. 2019 Apr 25;14(4):e0215907. doi: 10.1371/journal.pone.0215907 (PMC6483347; doi:10.1371/journal.pone.0215907)
Supplement: S1 File — (DOCX) [file pone.0215907.s001.docx]

Table A. Association between age and depression caseness and the rural-urban difference among men with non-missing BMI at Wave 1

|  | **Model 1** | **Model 2** | **Model 3** | **Model 4** |
| --- | --- | --- | --- | --- |
|  | OR (95% CI) | OR (95% CI) | OR (95% CI) | OR (95% CI) |
| **Age** (centred at 45 years) | 1.02 (0.98,1.06) | 1.00 (0.97,1.04) | 1.00 (0.96,1.03) | 0.99 (0.95,1.03) |
| **Age^2^** (centred at 45 years) | 1.00 (1.00,1.00) | 1.00 (1.00,1.00) | 1.00 (1.00,1.00) | 1.00 (1.00,1.00) |
| **Urbanisation** |  |  |  |  |
| Rural | Ref | Ref | Ref | Ref |
| Semi-urban | 0.58 (0.34,0.99) | 0.62 (0.37,1.04) | 0.64 (0.39,1.05) | 0.64 (0.39,1.06) |
| Urban | 0.49 (0.28,0.86) | 0.67 (0.39,1.16) | 0.70 (0.41,1.20) | 0.64 (0.37,1.10) |
| **Region** |  |  |  |  |
| East |  | Ref | Ref | Ref |
| Central |  | 1.71 (1.43,2.04) | 1.68 (1.41,1.99) | 1.54 (1.30,1.83) |
| Northeast |  | 1.05 (0.78,1.42) | 1.10 (0.81,1.48) | 0.95 (0.70,1.27) |
| West |  | 1.87 (1.57,2.22) | 1.77 (1.50,2.10) | 1.70 (1.44,2.01) |
| **Education** |  |  |  |  |
| No formal education |  | Ref | Ref | Ref |
| Primary |  | 0.81 (0.69,0.97) | 0.84 (0.71,0.99) | 0.88 (0.74,1.03) |
| Lower secondary |  | 0.73 (0.60,0.88) | 0.75 (0.63,0.90) | 0.81 (0.67,0.97) |
| Upper secondary & higher |  | 0.49 (0.38,0.63) | 0.51 (0.40,0.65) | 0.55 (0.44,0.71) |
| **Retirement** |  |  |  |  |
| No |  | Ref | Ref | Ref |
| Yes |  | 1.33 (1.12,1.57) | 1.31 (1.12,1.54) | 1.13 (0.95,1.33) |
| Missing |  | 2.65 (1.25,5.64) | 2.82 (1.36,5.86) | 2.39 (1.13,5.05) |
| **Marital status** |  |  |  |  |
| Married |  | Ref | Ref | Ref |
| Unmarried |  | 1.63 (1.34,1.98) | 1.66 (1.39,2.00) | 1.71 (1.42,2.06) |
| **Household amenities** |  |  |  |  |
| 0-2 |  | Ref | Ref | Ref |
| 3-4 |  | 0.75 (0.63,0.89) | 0.77 (0.66,0.90) | 0.80 (0.68,0.94) |
| 5-6 |  | 0.49 (0.40,0.59) | 0.52 (0.43,0.62) | 0.55 (0.46,0.66) |
| ≥7 |  | 0.36 (0.28,0.45) | 0.39 (0.31,0.48) | 0.42 (0.34,0.53) |
| Missing |  | 0.54 (0.22,1.32) | 0.56 (0.24,1.31) | 0.54 (0.23,1.29) |
| **Social activity** |  |  |  |  |
| No |  |  | Ref | Ref |
| Less often |  |  | 1.00 (0.85,1.17) | 1.03 (0.87,1.21) |
| Almost every week |  |  | 0.72 (0.60,0.88) | 0.75 (0.62,0.91) |
| Almost daily |  |  | 0.71 (0.61,0.83) | 0.75 (0.64,0.88) |
| **Alcohol drinking** |  |  |  |  |
| No |  |  | Ref | Ref |
| <1/month |  |  | 0.75 (0.61,0.92) | 0.77 (0.62,0.94) |
| 1-3/month |  |  | 0.85 (0.67,1.07) | 0.84 (0.66,1.07) |
| 1-6/week |  |  | 0.82 (0.67,1.02) | 0.83 (0.67,1.03) |
| ≥1/day |  |  | 0.67 (0.57,0.79) | 0.70 (0.60,0.83) |
| Missing |  |  | 0.67 (0.49,0.93) | 0.67 (0.48,0.93) |
| **Smoking** |  |  |  |  |
| Never |  |  | Ref | Ref |
| Former |  |  | 1.20 (0.99,1.46) | 1.14 (0.93,1.39) |
| Current |  |  | 1.23 (1.03,1.45) | 1.21 (1.02,1.43) |
| Missing |  |  | 1.00 (0.78,1.29) | 0.99 (0.76,1.28) |
| **BMI** |  |  |  |  |
| >15 & ≤18.5 |  |  | 1.53 (1.22,1.92) | 1.50 (1.18,1.90) |
| >18.5 & ≤24 |  |  | Ref | Ref |
| >24 & ≤28 |  |  | 0.88 (0.76,1.03) | 0.84 (0.72,0.97) |
| >28 |  |  | 0.73 (0.57,0.93) | 0.68 (0.53,0.87) |
| **Number of ADLs** |  |  |  |  |
| 0 |  |  |  | Ref |
| 1 |  |  |  | 2.77 (2.29,3.35) |
| 2+ |  |  |  | 5.90 (4.71,7.39) |
| Missing |  |  |  | 0.63 (0.27,1.48) |
| **Age × Urbanisation** |  |  |  |  |
| Age × semi-urban | 1.02 (0.95,1.09) | 1.03 (0.96,1.10) | 1.03 (0.96,1.10) | 1.04 (0.97,1.11) |
| Age × urban | 0.98 (0.92,1.05) | 0.98 (0.91,1.05) | 0.98 (0.92,1.05) | 1.01 (0.95,1.08) |
| Age^2^ × semi-urban | 1.00 (1.00,1.00) | 1.00 (1.00,1.00) | 1.00 (1.00,1.00) | 1.00 (1.00,1.00) |
| Age^2^ × urban | 1.00 (1.00,1.00) | 1.00 (1.00,1.00) | 1.00 (1.00,1.00) | 1.00 (1.00,1.00) |
| **Period** |  |  |  |  |
| Wave 1 | Ref | Ref | Ref | Ref |
| Wave2 | 0.92 (0.70,1.21) | 1.14 (0.87,1.50) | 1.28 (0.96,1.70) | 1.20 (0.89,1.61) |
| Wave3 | 0.86 (0.64,1.16) | 1.13 (0.84,1.52) | 1.13 (0.85,1.52) | 1.08 (0.80,1.46) |
| **Age × Period** |  |  |  |  |
| Age×Wave2 | 0.98 (0.97,1.00) | 0.98 (0.97,0.99) | 0.98 (0.96,0.99) | 0.98 (0.97,1.00) |
| Age×Wave3 | 1.00 (0.98,1.01) | 0.99 (0.97,1.01) | 0.99 (0.97,1.00) | 0.99 (0.97,1.00) |

OR: odds ratio; CI: confidence interval; Ref: reference category

Table B. Association between age and depression caseness and the rural-urban difference among women with non-missing BMI at Wave 1

|  | **Model 1** | **Model 2** | **Model 3** | **Model 4** |
| --- | --- | --- | --- | --- |
|  | OR (95% CI) | OR (95% CI) | OR (95% CI) | OR (95% CI) |
| **Age** (centred at 45 years) | 1.06 (1.03,1.08) | 1.03 (1.00,1.05) | 1.03 (1.00,1.05) | 1.02 (0.99,1.05) |
| **Age^2^** (centred at 45 years) | 1.00 (1.00,1.00) | 1.00 (1.00,1.00) | 1.00 (1.00,1.00) | 1.00 (1.00,1.00) |
| **Urbanisation** |  |  |  |  |
| Rural | Ref | Ref | Ref | Ref |
| Semi-urban | 0.61 (0.42,0.87) | 0.74 (0.52,1.05) | 0.72 (0.51,1.03) | 0.75 (0.53,1.07) |
| Urban | 0.27 (0.18,0.42) | 0.41 (0.27,0.64) | 0.40 (0.26,0.62) | 0.43 (0.28,0.67) |
| **Region** |  |  |  |  |
| East |  | Ref | Ref | Ref |
| Central |  | 1.67 (1.45,1.92) | 1.66 (1.45,1.92) | 1.54 (1.34,1.78) |
| Northeast |  | 1.38 (1.11,1.73) | 1.34 (1.07,1.68) | 1.25 (1.00,1.57) |
| West |  | 1.92 (1.67,2.20) | 1.88 (1.64,2.16) | 1.77 (1.54,2.03) |
| **Education** |  |  |  |  |
| No formal education |  | Ref | Ref | Ref |
| Primary |  | 0.80 (0.69,0.93) | 0.81 (0.70,0.94) | 0.84 (0.72,0.97) |
| Lower secondary |  | 0.63 (0.53,0.74) | 0.64 (0.54,0.76) | 0.67 (0.57,0.80) |
| Upper secondary & higher |  | 0.54 (0.42,0.69) | 0.55 (0.43,0.70) | 0.56 (0.44,0.71) |
| **Retirement** |  |  |  |  |
| No |  | Ref | Ref | Ref |
| Yes |  | 1.17 (1.04,1.32) | 1.21 (1.12,1.41) | 1.09 (0.97,1.23) |
| Missing |  | 1.40 (0.64,3.07) | 1.39 (0.63,3.07) | 1.05 (0.47,2.32) |
| **Marital status** |  |  |  |  |
| Married |  | Ref | Ref | Ref |
| Unmarried |  | 1.20 (1.05,1.38) | 1.21 (1.05,1.39) | 1.21 (1.05,1.38) |
| **Household amenities** |  |  |  |  |
| 0-2 |  | Ref | Ref | Ref |
| 3-4 |  | 0.89 (0.77,1.03) | 0.90 (0.78,1.04) | 0.94 (0.82,1.09) |
| 5-6 |  | 0.67 (0.58,0.78) | 0.69 (0.59,0.81) | 0.73 (0.63,0.85) |
| ≥7 |  | 0.50 (0.42,0.60) | 0.51 (0.43,0.62) | 0.55 (0.46,0.66) |
| Missing |  | 0.91 (0.54,1.55) | 0.91 (0.53,1.55) | 0.93 (0.55,1.59) |
| **Social activity** |  |  |  |  |
| No |  |  | Ref | Ref |
| Less often |  |  | 1.07 (0.92,1.23) | 1.08 (0.93,1.24) |
| Almost every week |  |  | 0.83 (0.70,0.98) | 0.84 (0.71,0.99) |
| Almost daily |  |  | 0.76 (0.67,0.86) | 0.80 (0.71,0.90) |
| Missing |  |  | 2.21 (0.17,29.67) | 2.56 (0.16,39.78) |
| **Alcohol drinking** |  |  |  |  |
| No |  |  | Ref | Ref |
| Yes |  |  | 1.15 (0.99,1.34) | 1.14 (0.95,1.29) |
| Missing |  |  | 1.42 (0.91,2.20) | 1.41 (0.91,1.76) |
| **Smoking** |  |  |  |  |
| Never |  |  | Ref | Ref |
| Former/current |  |  | 1.26 (1.03,1.54) | 1.18 (0.97,1.44) |
| Missing |  |  | 1.09 (0.68,1.74) | 1.10 (0.69,1.76) |
| **BMI** |  |  |  |  |
| >15 & ≤18.5 |  |  | 1.21 (0.97,1.49) | 1.19 (0.96,1.47) |
| >18.5 & ≤24 |  |  | Ref | Ref |
| >24 & ≤28 |  |  | 0.95 (0.84,1.06) | 0.92 (0.82,1.04) |
| >28 |  |  | 0.86 (0.74,1.01) | 0.76 (0.65,0.89) |
| **Number of ADLs** |  |  |  |  |
| 0 |  |  |  | Ref |
| 1 |  |  |  | 2.19 (1.88,2.54) |
| 2+ |  |  |  | 4.94 (4.14,5.90) |
| Missing |  |  |  | 0.54 (0.28,1.02) |
| **Age × Urbanisation** |  |  |  |  |
| Age × semi-urban | 0.99 (0.95,1.04) | 0.99 (0.95,1.04) | 0.99 (0.95,1.04) | 1.00 (0.95,1.05) |
| Age × urban | 1.04 (0.98,1.10) | 1.03 (0.97,1.09) | 1.04 (0.98,1.10) | 1.05 (0.99,1.11) |
| Age^2^ × semi-urban | 1.00 (1.00,1.00) | 1.00 (1.00,1.00) | 1.00 (1.00,1.00) | 1.00 (1.00,1.00) |
| Age^2^ × urban | 1.00 (1.00,1.00) | 1.00 (1.00,1.00) | 1.00 (1.00,1.00) | 1.00 (1.00,1.00) |
| **Period** |  |  |  |  |
| Wave 1 | Ref | Ref | Ref | Ref |
| Wave2 | 1.03 (0.84,1.27) | 1.19 (0.97,1.47) | 1.21 (0.98,1.49) | 1.19 (0.96,1.46) |
| Wave3 | 1.12 (0.90,1.39) | 1.38 (1.10,1.72) | 1.38 (1.10,1.72) | 1.38 (1.10,1.72) |
| **Age × Period** |  |  |  |  |
| Age×Wave2 | 0.98 (0.97,0.99) | 0.98 (0.97,0.99) | 0.98 (0.97,0.99) | 0.98 (0.97,0.99) |
| Age×Wave3 | 0.99 (0.98,1.00) | 0.98 (0.97,1.00) | 0.99 (0.97,1.00) | 0.98 (0.97,0.99) |

OR: odds ratio; CI: confidence interval; Ref: reference category

| 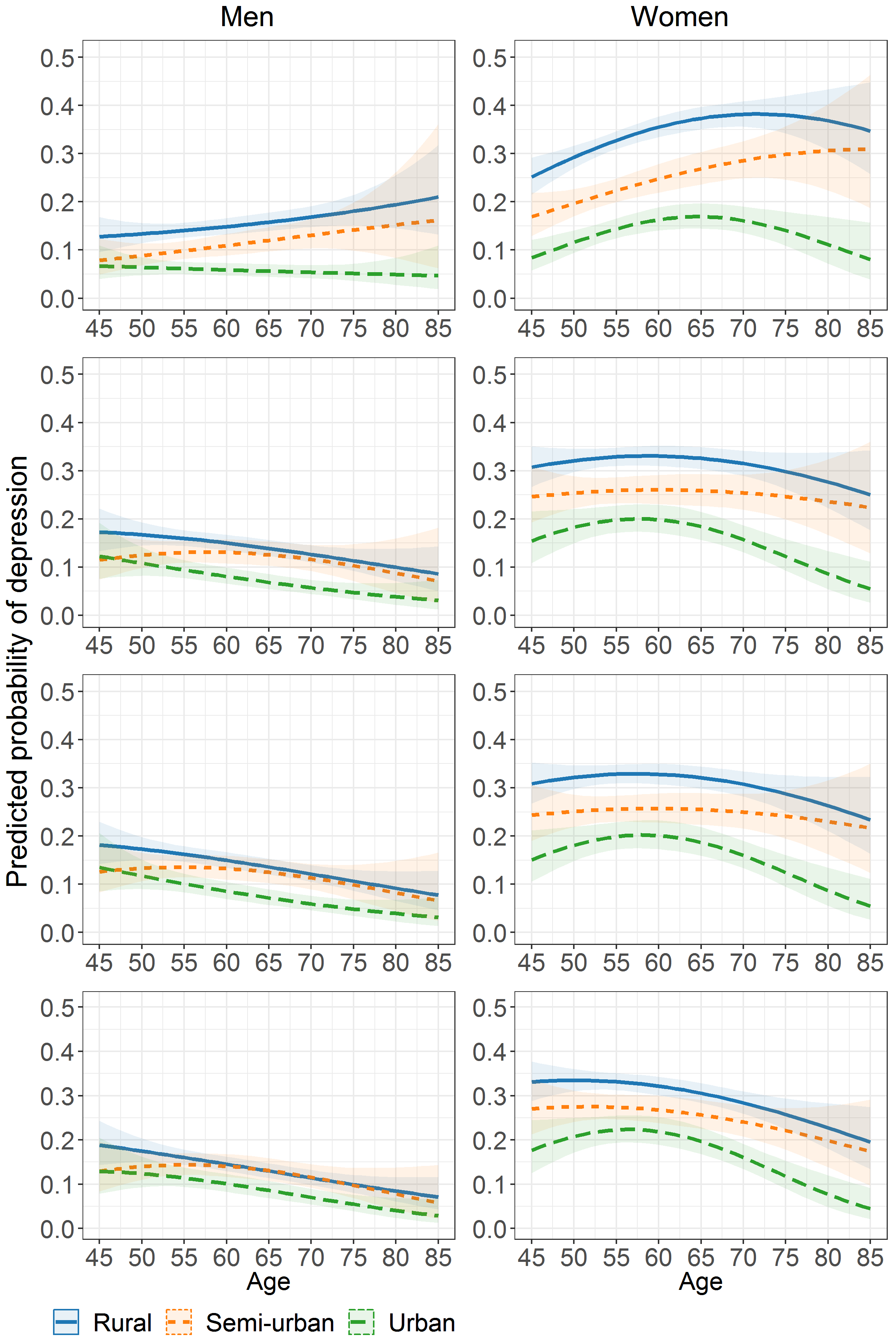 | **Model 1** |
| --- | --- |
|  | **Model 2** |
|  | **Model 3** |
|  | **Model 4** |

Figure A. Rural-urban difference in age trajectories of marginal predicted probability of depression caseness among participants with BMI non-missing at Wave 1 (inverse probability weighted)
